# Assessing the Crop-Water Status in Almond (*Prunus dulcis* Mill.) Trees via Thermal Imaging Camera Connected to Smartphone

**DOI:** 10.3390/s18041050

**Published:** 2018-03-31

**Authors:** Iván Francisco García-Tejero, Carlos José Ortega-Arévalo, Manuel Iglesias-Contreras, José Manuel Moreno, Luciene Souza, Simón Cuadros Tavira, Víctor Hugo Durán-Zuazo

**Affiliations:** 1 Instituto Andaluz de Investigación y Formación Agraria y Pesquera (IFAPA), Centro “Las Torres-Tomejil”, Ctra. Sevilla-Cazalla km. 12,2, 41.200 Alcalá del Río (Sevilla), Spain; carorca6@gmail.com (C.J.O.-A.); m.iglesiascontreras@gmail.com (M.I.-C.); josemanuel.moreno89@gmail.com (J.M.M.); luciene.sf05@yahoo.com.br (L.S.); victorh.duran@juntadeandalucia.es (V.H.D.-Z.); 2 Universidade Estadual do Norte Fluminense Darcy Ribeiro (UENF), Av. Alberto Lamego, 2000 Campos dos Goytacazes, RJ, Brazil; 3 Departamento de Ingeniería Forestal, Universidad de Córdoba, Campus de Rabanales, E-14071 Córdoba, Spain; scuadros@uco.es

**Keywords:** thermal readings, irrigation scheduling, data acquisition, thermal indexes, non-water stressed baselines

## Abstract

Different tools are being implemented in order to improve the water management in agricultural irrigated areas of semiarid environments. Thermography has been progressively introduced as a promising technique for irrigation scheduling and the assessing of crop-water status, especially when deficit irrigation is being implemented. However, an important limitation is related to the cost of the actual cameras, this being a severe limitation to its practical usage by farmers and technicians. This work evaluates the potential and the robustness of a thermal imaging camera that is connected to smartphone (Flir One) recently developed by Flir Systems Inc. as a first step to assess the crop water status. The trial was developed in mature almond (*Prunus dulcis* Mill.) trees that are subjected to different irrigation treatments. Thermal information obtained by the Flir One camera was deal with the thermal information obtained with a conventional Thermal Camera (Flir SC660) with a high resolution, and subsequently, confronted with other related plant physiological parameters (leaf water potential, Ψ_leaf_, and stomatal conductance, g_s_). Thermal imaging camera connected to smartphone provided useful information in estimating the crop-water status in almond trees, being a potential promising tool to accelerate the monitoring process and thereby enhance water-stress management of almond orchards.

## 1. Introduction

In semiarid environments, such as the Mediterranean basin, the irrigated agriculture is seriously affected by the scarcity and irregularity of water resources availability, and worsened with a progressive climate change incidence [1,2]. According to the Intergovernmental Panel on Climate Change IPCC [3], this fact is promoting scenarios of water-resources depletions, with more severe periods of lower rainfall during the wet periods, and more pronounced hot waves during the maximum evapotranspirative demand period. Thus, the crop-water demand is expected to increase (30% as compared to current consumption), promoting an imbalance between the irrigation-water demand and the available resources [4].

Under this scenario, it is crucial to adapt different strategies and methodologies to increase the irrigation water productivity, stablishing the best strategies for an efficient and sustainable water management [5,6].

Deficit irrigation (DI) strategies have been traditionally used in many arid and semi-arid areas of Mediterranean countries, where the available water resources are not sufficient to cover the crops water demand, although they not always have been properly applied. For this reason, a wide number of DI strategies in different crops has been applied, with the aim of increasing the irrigation water-use efficiency, keeping the final yield values, and achieving significant water savings [7]. However, the application of these strategies requires properly assessing the crop-water status, to ensure the fitting crop development without compromising the yield, especially when water-stress is applied in different crop phenological stages [8]. 

Traditionally, crop-water monitoring has been developed by means of punctual measurements of stem or leaf water potential (Ψ_leaf_ or Ψ_steam_) at midday or pre-dawn [9,10], or monitoring the gas-exchange parameters, such as transpiration, stomatal conductance (g_s_), or net photosynthetic rate [11].

On the other hand, infrared thermography images are being progressively introduced to monitor the crop-water status in woody crops [12].

Many works have been developed, relating changes on canopy temperature (T_C_) and water stress situations, in which, this technique has been properly described as a promising methodology for crop-water monitoring in different woody crops, such as citrus [13,14], young almonds [15], vines [12], or olives [16]. In addition, T_C_ could be considered as a good source of information in estimating the g_s_, and plant-water status [17,18], although the relationships between leaf/canopy temperature and crop physiological parameters are not always straightforward, especially in the field due to the large variation of weather variables (air temperature, solar radiation, the angle of incident radiation, wind speed, and vapour pressure deficit) [15,17]. 

With the aim of minimizing the effects of environmental factors, normalizing their variation, and establishing a simple methodology to quantify the level of crop-water status, there are different thermal indexes that can be estimated and implemented for a proper explanation. In this sense, Idso et al. [19] evidenced high differences in temperature values between the canopy and air temperature in plants under water stress, while these differences were very low (even negative) in well-irrigated plants, defining the difference between canopy and air temperature as a simple thermal index. García-Tejero et al. [12,13,15,16] found significant relationships between this thermal index and some physiological parameters, such Ψ_leaf_ or g_s_ in citrus, almonds, vines, or olives, among others. 

By the contrast, other thermal indices relate the actual canopy temperature to the temperature of selected reference surfaces, usually ‘dry’ and ‘wet’ references; which mimics the canopy temperatures when stomata are fully closed and open, respectively. These thermal stress indices have been widely and successfully applied in many works for monitoring the crop-water status under deficit irrigation systems [12,16,18]. 

The main constraints of this technique are focused in the correct interpretation of the infrared thermal information, the convenient moment and strategy to take the readings, and the most representative thermal index to define the crop-water status. 

In this work, we hypothesize that thermal information that is provided by the low-cost thermal camera “Flir One” connected to a smartphone is enough robust and feasible to assess the crop-water status for agricultural crops. Thus, the objective of the present experiment was to evaluate and compare the feasibility in field readings of low-cost thermal camera (T_SPH_) in relation to a conventional Thermal Imaging Camera (T_TIC_) in an experimental almond (*Prunus dulcis* Mill) orchards that were subjected to deficit irrigation regimes. (ThermaCam Flir SC660). Additionally, there were defined the non-water stressed baselines for almond crop, and the main relationships with plant physiological parameters (Ψ_leaf_ and g_s_).

## 2. Material and Methods

### 2.1. Experimental Site

The trial was conducted during 2017, from 152 to 212 day of the year (DOY) in an experimental orchard of almond (*Prunus dulcis* Mill. D.A. Webb cv. Guara, grafted onto GF677), which was located in the Guadalquivir river basin (37°30′47′′ N; 5°58′2′′ O) (Seville, SW Spain). Trees were planted in 2009, spaced 6 m × 7 m, and drip irrigated using two pipe lines with emitters of 2.3 L·h^−1^, and 14 emitters per tree.

The soil is silty loam, typical Fluvisol, 2.5 m deep, fertile, and organic matter content <15.0 g·kg^−1^. Roots are located predominately in the first 50 cm of soil, corresponding to the intended wetting depth, although these exceed more than one meter in depth. Soil-water content values at field capacity (−0.33 MPa) and permanent wilting point (−1.5 MPa) are 0.35 and 0.12 m^3^·m^–3^, respectively, with an allowable soil-water depletion level of 0.27 m^3^·m^–3^.

The climatology in the study area is attenuated meso-Mediterranean, with an annual ET_0_ rate of 1400 mm and accumulated rainfall of 540 mm, which was mainly distributed from October to April.

The total rainfall and evapotranspiration registered during the experimental period were 0.4 and 387 mm, respectively. Daily maximum temperatures ranged between 28.7 and 48.7 °C, whereas the daily average temperature ranged between 22.2 and 32 °C. Regarding to the daily average relative humidity, these values ranged between 32.2 and 67.3%. 

### 2.2. Irrigation Treatments

Three irrigation treatments were defined: (i) a full irrigated as a control treatment (FI), which received 100% of the crop evapotranspiration (ET_C_) during the irrigation period; (ii) a sustained deficit irrigation (SDI_50_), which received 100% of ET_C_, except during the kernel filling period and pre-harvest, when this treatment was irrigated with ~50% of ET_C_; and, (iii) and a low-frequency deficit irrigation (LFDI), which received the 100% ET_C_ during the irrigation period, except during the kernel-filling stage and pre-harvest; when this treatment was irrigated according the registered values of Ψ_leaf_ measured in shaded leaves. That is, during the kernel-filling period, this treatment was subjected to irrigation-restriction cycles with the following irrigation dynamic: once started the kernel-filling period, irrigation was suppressed, until reaching values of Ψ_leaf_ that were close to −2.0 MPa. Then, trees were re-watered with the same periodicity and amount of water as FI (approximately during 7–10 days) until reaching similar values of Ψ_leaf_ to those registered in FI (~−1.5 MPa). Once this threshold value was reached, this treatment was subjected to a new restriction period until the threshold of Ψ_leaf_ (~−2.0 MPa) was again surpassed. This dynamic of irrigation-restriction cycles was maintained during the whole stage of kernel filling period until harvesting.

Irrigation doses were calculated according to the methodology proposed by Allen et al. [20], obtaining the values of reference evapotranspiration (ET_0_) by using a weather station that was installed in the same experimental orchard. The local crop coefficients used during the irrigation period ranged between 1.0 and 1.2, according to the results that were obtained by García-Tejero et al. [21]. 

### 2.3. Experimental Design and Plant Measurements

The experimental design was of randomized blocks, with four replications per irrigation treatment. Each replication had 15 trees (three rows and five trees per row) being monitored the three central rows for each replication (*n* = 12). 

During the kernel filling period, when water restrictions were applied, crop-water monitoring was done throughout the measurements of leaf water potential (Ψ_leaf_) the stomatal conductance to water vapour (g_s_) and canopy temperature (T_C_). These readings were taken between 12:00 and13:30 GTM, in 12 trees per irrigation treatment and with a periodicity of 7–10 days. 

Measurements of Ψ_leaf_ were developed by using a pressure chamber (Soil Moisture Equipment Corp., Sta. Barbara, CA, USA), monitoring 12 trees per irrigation treatment (one leaf per tree), which was located in the north side of the tree and being totally mature, fresh, and shaded, at 1.5 m of height, approximately. 

Additionally, in these same trees, it was measured the stomatal conductance to water vapour (g_s_), using a porometer SC-1 (Decagon Devices, INC, Pullman, WA, USA), these measurements being done in one leaf completely exposed to the sun per monitored tree, and at 1.5 m of height.

The T_C_ was measured by thermal imaging. Measurements were made at the same time of the remaining measurements, by using two different cameras: (i) a ThermaCam (Flir SC660, Flir Systems, USA, 7–13 μm, 640 × 480 pixels), using an emissivity (ε) set at 0.95 (T_TIC_); and, (ii) a low-cost ThermaCam (Flir One, Flir Systems, Wilsonville, OR, USA). This camera uses a thermal sensor (8–14 μm, 80 × 60 pixels) and a digital sensor (1440 × 1080 pixels) that was connected to a smartphone (T_SPH_). This fact allows for overlapping two images (a false-coloured with a digital image) and enabling the subsequent analysis. For this camera, a matt emissivity was used (ε = 0.95), this being the same as the used for ThermaCam Flir SC660, and it has been suggested by Costa et al. [22]. More details about this thermal camera can be found in the official webpage of the manufacturer (http://www.flir.es/flirone/ios-android/). In both of the cameras, each pixel corresponds to an effective temperature reading. Taking into account the different resolution and field of view for each camera, those colored areas that correspond to the same place of the surface canopy were selected. The images were taken in the sunlit side of the trees, as it has been suggested in previous works in order to identify the highest differences between irrigation treatments [16,19,22], with the imager being placed at 2 m of the canopy. 

**Figure 1:**
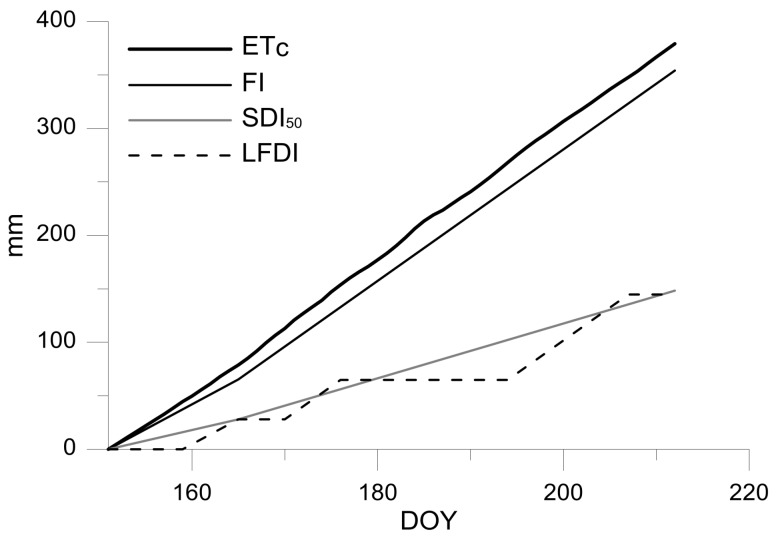
Accumulated crop evapotranspiration (ET_C_) and irrigation doses applied. FI, full irrigated at 100% ET_C_; SDI_50_, sustained deficit irrigation at 50% of ET_C_; and, LFDI, low-frequency deficit irrigation.

Images were analyzed using two different cameras: for the case of the images taken with the thermal camera Flir SC660, these were analyzed by using the Flir Research Pro, which allows fpr selecting different areas of the image (in our case 3–4 sunlit areas within the same image) (Figure 1). By contrast, for the case of the images taken with the Flir One camera, these were captured by using the free software “Flir One” for system; and subsequently, these were analyzed by using the Flir Tools application, for Android smartphone (Figure 1). This application allows the operator to select different areas of the same image, and analyze them, overlapping the digital and false-coloured image, which facilitates the selection of the most representative sunlit areas, although the accuracy is lower than provided by the Flir Research Pro.

When considering the T_C_ values that were obtained at tree level, two different thermal indices were calculated: the difference between canopy and the surrounding air (ΔT_canopy-air_) and the crop water stress index (CWSI), these being calculated according to Costa et al. [22], as follows:

ΔT_canopy-air_ = T_C_ − T_air_ (1)

(2) $$\mathrm{CWSI}=\frac{\Delta T_{\mathrm{canopy-air}}-\Delta \mathrm{Twet}}{\Delta \mathrm{Tdry}-\Delta \mathrm{Twet}}$$

where ΔT_canopy-air_, ΔT_dry_, and ΔT_wet_ are the differences between canopy and air temperature for the crop in the moment of the measurement, when the crop has the stomata fully closed and when is fully transpiring, respectively. T_C_ is the canopy temperature and T_air_ the temperature of the surrounding air.

To obtain the reference values of ΔT_wet_, there was estimated the non-water stress baseline (ΔT_canopy-air_ = a + b × VPD), according to Idso et al. [19], using a ΔT_dry_ value that is equal to 5 °C, as it was proposed by Jackson et al. [23]. Non-water stress baseline was defined using the canopy temperature readings obtained from fully irrigated trees.

Additionally, T_air_ and RH (both necessary to estimate the VPD for each monitoring point) were obtained by using a temperature-humidity-instrument (H560 Dewpoint Pro, ProfiLab24 GmbH, Berlin, Germany).

### 2.4. Statistical Analysis

For each measurement day, an exploratory descriptive analysis of data (Ψ_leaf_, g_s_, and T_C_), applying a Levene’s test to check the variance homogeneity of the studied variables was made. Significant differences between irrigation treatments (*p* ≤ 0.05) in the studied variables were analyzed by applying a one-way ANOVA and a Tukey’s test for treatment separation, with the SPSS statistical software (SPSS Inc., 15.0 Statistical package, Chicago, IL, USA). 

To evaluate the precision of measurements taken by the Flir One sensor, a linear correlation analysis between T_TIC_ and T_SPH_ was performed (*n* = 162). 

Once defined, the precision measurements taking by using the Flir One sensor, the non-water stressed baselined for both sources of thermal information were obtained. The obtained functions were compared between them (slope and intercept) using a covariance analysis at a confidence level of 95%.

Finally, there were obtained the relationships different thermal indexes that were obtained from the measurements provided by the Flir One sensor, and the monitored physiological variables, throughout a linear correlation analysis (*p* < 0.05).

## 3. Results

### 3.1. Weather and Plant Physiological Parameters

**Figure 2:**
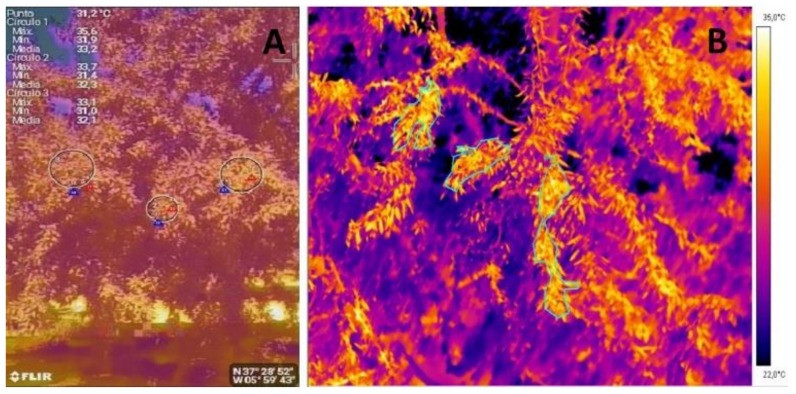
False colored images taken by using ThermaCam (Flir One, Flir Systems, USA) connected to a smartphone (**A**) and with a conventional ThermaCam (Flir SC660, Flir Systems, USA) (**B**).

Figure 2 shows the accumulated ET_C_ and the irrigation that is applied for each treatment during the studied period. In this regard, control treatment received during the kernel filling period 355 mm, whereas SDI_50_ and LFDI received 148 mm, which represented 58% less water than the control trees.

**Figure 3:**
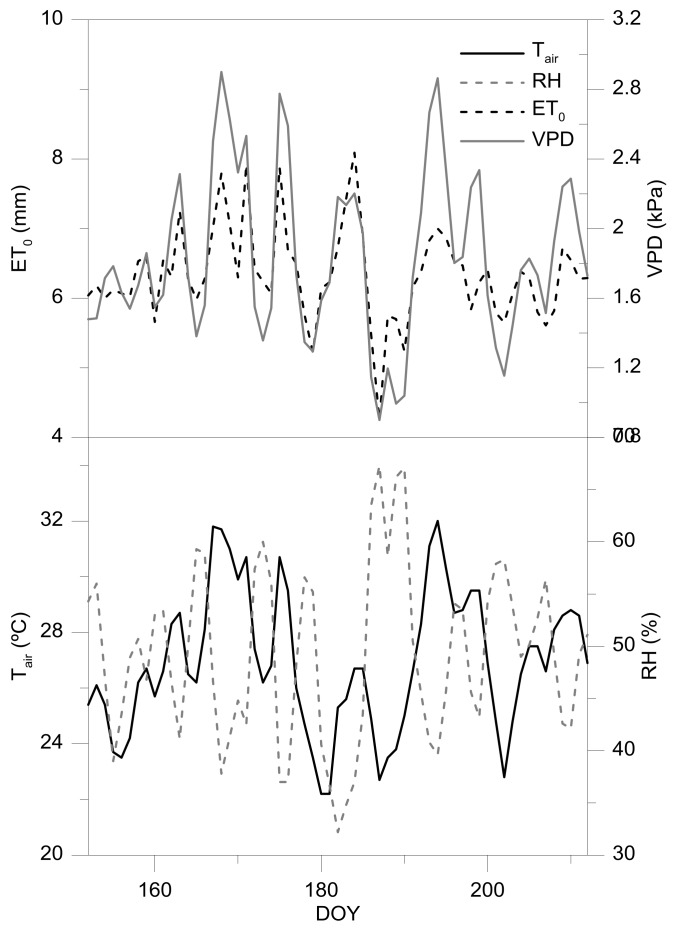
Weather conditions during the experimental period. T_air_, average air temperature; RH, relative humidity; ET_0_ evapotranspiration; VPD, vapour pressure deficit.

The dynamics of average weather conditions during the monitoring period displays in Figure 3. The T_air_ and RH showed an inverse trend, thus those days in which there were reached the maximum values of T_air_, these coincided with those with the lowest values of RH. Moreover, during these days, the maximum values of ET_0_ and VPD were reached. That is, on some days, the values of ET_0_ higher than 7 mm·day^−1^ with average VPD up to 2.5 kPa were reached.

**Figure 4:**
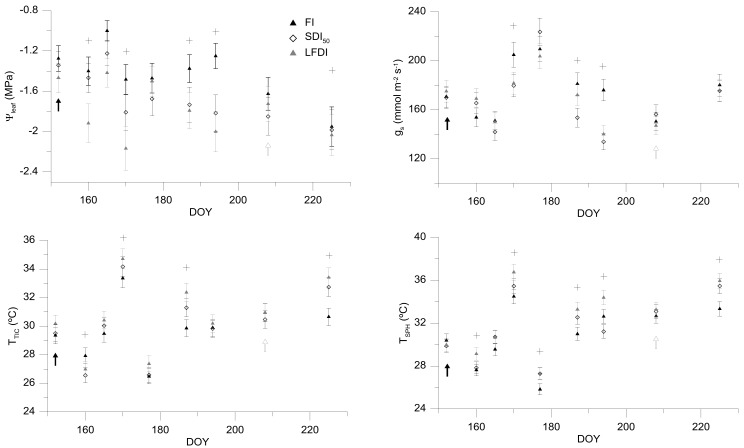
Temporal dynamics of leaf water potential (Ψ_leaf_); stomatal conductance (g_s_), canopy temperature readings with a Flir SC660 camera (T_TIC_); and with a Flir One camera connected to a smartphone (T_SPH_). FI, Full irrigated treatment at 100% ET_C_; SDI_50_, sustained deficit irrigation treatment at 50% ET_C_; LFDI, low-frequency deficit irrigation, irrigated in terms of Ψ_leaf_ values. Black arrows correspond to the beginning of water stress period, white arrows to the moment in which irrigation is suppressed in all of the treatments (seven days before the harvesting). The asterisks show those moments in which significant differences between FI and stressed treatments were reached. Vertical lines represent the standard error for each value.

During the measuring days there were monitored different variables in order to monitor the crop-water status Ψ_leaf_ and g_s_, showing a trend according to irrigation strategies applied. At the beginning of the experiment, all of the monitored trees had a similar physiological status, with values of Ψ_leaf_ around −1.3 MPa, and g_s_ around 170 mmol m^−2^·s^−1^ (Figure 4). Once the deficit irrigation strategies were established, the registered values in the different parameters were changing according to the irrigation water amount applied in each treatment. In this line, FI showed values of Ψ_leaf_ ranging between −1.0 and −1.6 MPa, whereas, the SDI_50_ showed a descending trend, reaching the most negative values between −1.8 and −2.0 MPa. Finally, LFDI treatment showed a very interesting trend, reaching the most negative values (close to −2.0 MPa, even lower than this), those days in which the treatment had been subjected to 7–10 days of irrigation withholding; and once irrigated during 7–10 days with the same irrigation strategy that FI, its values of Ψ_leaf_ were similar to those that were reported by the FI.

By the contrast, the values of g_s_ were more variable than Ψ_leaf_. These values were more influenced by the weather conditions. In this agreement, the differences between treatments were not as clear as for the case of Ψ_leaf_, being very difficult to define the proper range of g_s_ in order to use this variable to define an optimum value.

### 3.2. Infrared Thermal Parameters: T_TIC_ vs. T_SPH_

**Figure 5:**
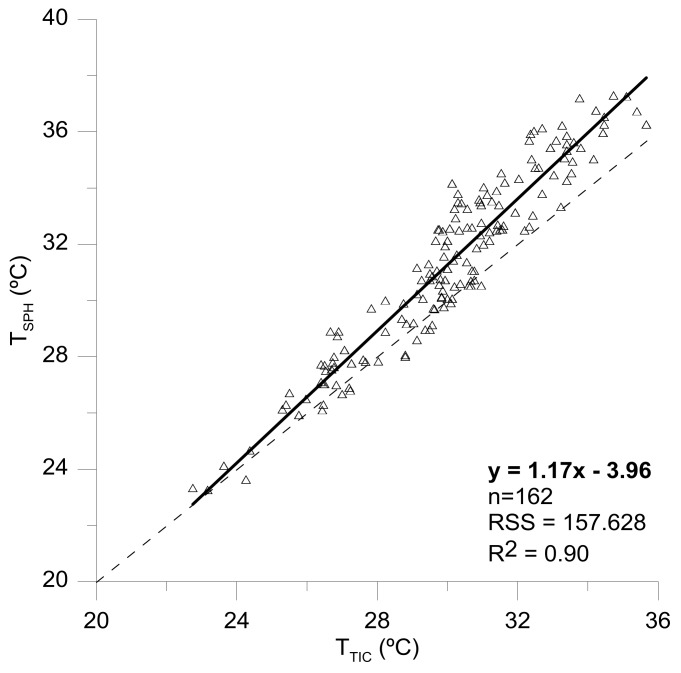
Linear relationship between the canopy temperature values obtained by using a Flir SC660 camera (T_TIC_); a Flir One camera connected to a smartphone (T_SPH_). Dotted line corresponds to the function *y* = *x*. RSS: Residual Sum of Squares.

The readings of canopy temperature by using the ThermaCam Flir SC660 (T_TIC_) and with the Flir One camera (T_SPH_) showed very similar trends, although the values of T_SPH_ were between 0.5 and 2 °C higher than those that were obtained with T_TIC_, especially for those higher T_TIC_ values (Figure 5). Nevertheless, the differences between treatments were more evident for the values of T_SPH_, in comparison to T_TIC_. Moreover, those days in which significant differences between treatments were reached in terms of Ψ_leaf_, similar differences were observed for the case of canopy temperature by using both of the thermal sensors.

Despite the differences in terms of absolute values of canopy temperature readings taken with both cameras, it was observed a very significant relationship between T_TIC_ and T_SPH_ (R^2^ = 0.90), suggesting that the obtained information between both sensors would be suitable to monitor the crop water status.

**Figure 6:**
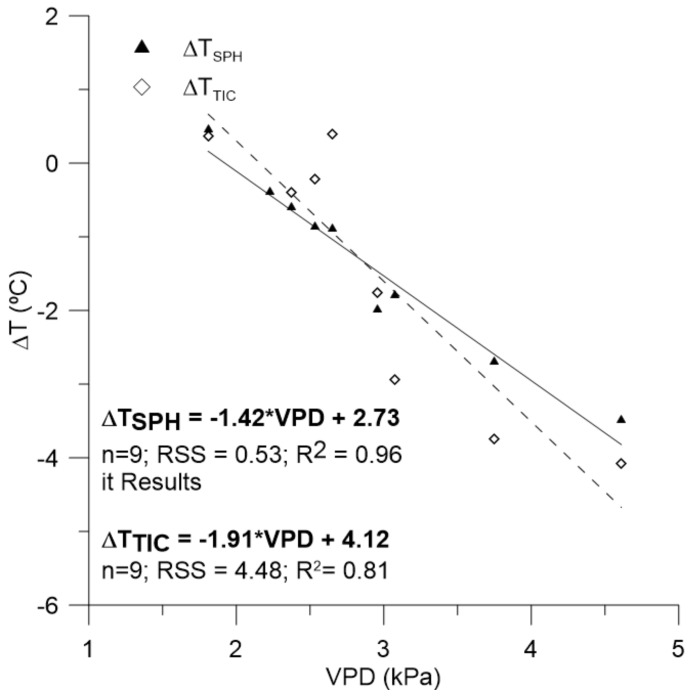
Non-water stressed baselines between the vapour pressure deficit (VPD) and the difference between the canopy and air temperature for both cameras Flir One thermal camera connected to smartphone (ΔT_SPH_) and a Flir SC660 camera (ΔT_TIC_).

When considering these results, there were obtained the water stress baselines, which define the relationships between the values of VPD in the moment of the reading and the difference between the canopy and air temperature (Figure 6). The obtained relationships were significant (*p* < 0.95) with R^2^ of 0.96 for the case of the differences related to the values of T_SPH_ (ΔT_SPH_) and R^2^ = 0.81 for the case of the differences related to the values of T_TIC_. By the contrast, the covariance analysis showed differences between these linear functions, being necessary a differential usage depending on the thermal camera.

**Figure 7:**
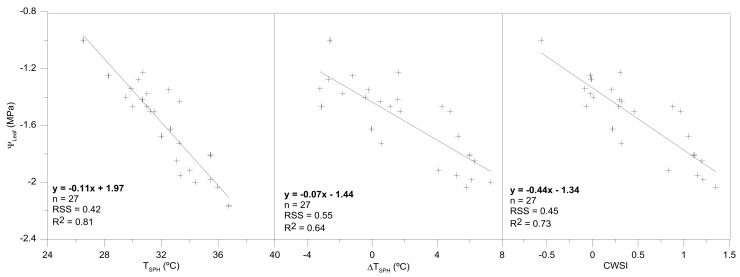
Relationships among different thermal parameters (T_SPH_; ΔT_SPH_) taken with Flir One thermal camera connected to smartphone; and the crop water stress index (CWSI)) and the leaf-water potential (Ψ_Leaf_). RSS: Residual Sum of Squares.

Taking into account that the obtained values of canopy temperature with the low-cost thermal sensor were sufficiently robust and representative of the crop-water status, the likeness with those that were obtained with the ThermaCam SC660; and, the robustness of the relationship between ΔT_SPH_ and VPD values; the relationships between different thermal indicators (T_SPH_; ΔT_SPH_ and the crop water stress index (CWSI) with the remaining physiological variables (Ψ_leaf_ and g_s_) were defined; in order to define the most representative of them, and the possibility of estimating the crop-water status exclusively by using thermal information (Figure 7). In this agreement, no significant relationships were obtained between the thermal indexes that were considered and g_s_ (not shown data), probably being promoted by the highly variability of g_s_ values, and the absence of higher differences between the treatments in relation to this physiological variable. By the contrast, significant relationships were obtained (*p* < 0.05) for the case of Ψ_leaf_ and thermal indexes. Even more, it is remarkable that the best results were obtained for the case of T_SPH_ in comparison to ΔT_SPH_ and CWSI; although between them, CWSI improved the results that were reported by the relationship between ΔT_SPH_ and VPD.

## 4. Discussion

In the last few years, new tools and different strategies to improve the irrigation water management are being demanded. In this context, there is an increasing interest in the use of remote sensing techniques to monitor crop water status, aiming to improve the irrigation scheduling.

To date, many works have been oriented to test the different strategies of image analysis and capturing, which have resulted in the development of effective protocols for field measurements based on the use of different thermal indices [16,24,25,26] and robust relationships between thermal information and plant physiological parameters. However, one of the main limitations that is linked to this technique is the necessity in comparing the obtained measurements with other physiological variables, in order to confirm if the information reported in terms of thermal data is in accordance to the crop-water status. That is, relationships between thermal and physiological data have been defined with the aim of corroborating if thermal information can be used to assess the crop-water status, and consequently, the irrigation scheduling. Overall, and taking into account the monitored physiological variables in this work, it can be assumed that Ψ_leaf_ was the parameter that reflected the highest differences between treatments, followed by the T_C_ that was measured with both thermal sensors. 

Another important question for being taken into account would be related to the higher variability in terms of g_s_ values. This fact was discussed by Eichi [27], reporting that g_s_ is a physiological measurement that is more affected by climatic conditions. For this reason, finding significant relationships between thermal indicators and this parameter was not possible, and something similar was reported by García-Tejero et al. [26] in mature almond trees, for measurements that were taken at midday. This constraint has been related to the anhisohydric pattern of almond when this crop is subjected to water stress situations. Other authors, such as Klein et al. [28] or Romero et al. [29], reported that under non-water stress conditions, g_s_ is less variable for particular conditions and values of vapour pressure deficit below to −2 KPa. However, during the experimental period, higher values of VPD were detected, and hence, this would have promoted a higher variability. 

Regarding the dynamics of T_C_ that are shown in Figure 4, and according to García-Tejero et al. [12], there are many variables, such as the air temperature, vapour pressure deficit, the radiation level, or the angle of incident on the leaf surface that will influence decisively the absolute value of T_C_, and must be considered. However, García-Tejero et al. [12,16] reported that the absolute values of T_C_ would be a proper indicator of crop physiological status, if thermal readings were taken within similar conditions, that is, at midday, taking these measurements in fully sunny exposed leaves and during the maximum evapotranspirative demand period (as it was done in this experiment). In spite of this, the use of absolute temperature to monitor the crop-water status could be difficult to take proper decisions if our objective is to schedule irrigation. In this regard, together with this thermal indicator, we evaluated two thermal indices, comparing their values with those that were reported by Ψ_leaf_ or g_s_ (Figure 6), being previously necessary to define the non-water stress baselines (Figure 5).

Previous works have demonstrated that thermography can be used to assess the crop water, although for a proper management of deficit irrigation strategies it is determinant to define the best thermal index to obtain the most robust information as possible. In this context, many reports have shown that best time of the day to do more robust and physiologically meaningful temperature readings was at midday [25,30]. When considering the simplicity and time-consuming aspects, the absolute value of canopy temperature would be recommendable because it is easy to calculate, as it has been reported in this work, high significant correlations can be obtained with Ψ_leaf_. In this agreement, this value has been successfully used in water stress monitoring of relevant woody crops, such as citrus, almonds, vines, or olives [12,13,16,31]. These works reported significant correlations of between T_C_ with Ψ_leaf_, when measurements were done at midday; which also emphasizes the physiological relevance of this “simpler” index.

A previous stage to transform the absolute values of T_C_ in a normalized thermal index (such as ΔT or CWSI) the knowledge of some climatic parameters as T_air_ and RH is necessary. This fact is determinant, being necessary to estimate these values under the same conditions in which the thermal measurements are being taken. In this line, García-Tejero et al. [12] reported that, if these variables are not monitored close to the trees that are being controlled it is possible to obtain worse relationships between the normalized thermal indexes than those are obtained for the absolute T_C_. The first constraint would be to define a proper non-water stressed baseline, which in our case, it was defined for the T_C_ readings taken with the ThermaCam Flir SC660 and the Flir One sensor. In our case, high significance relationships (*p* < 0.99) were obtained for both functions, although the obtained for the Flir One sensor resulted something better than the previous one. When comparing these functions (Figure 5) with others that were reported for this same crop, García-Tejero et al. [31] reported similar slopes (−1.88; −1.84; and −1.85), to the obtained for the function defined for the measurements taken with the ThermaCam Flir SC660 (the same that was used in this previous work). The main difference was that the measurements were taken along the day (from 8:00 to 20:00), whereas, in this work, this relationship was obtained by using measurements taken exclusively at midday. Likewise, these works imply an advance in the use of this technique to monitor the almond water status, not having evidence of other related works for this same crop.

In addition, Berni et al. [18], argued that the slope values for different functions could be affected by the errors in the estimation of T_C_ and the measurement of T_air_, although more interesting were the conclusions that were derived from these authors when they compared the effect of net radiation and wind speed in the interception point, suggesting that the slopes that were obtained for different non-water stressed baselines estimated from a theorist proposed model by them were very similar to those obtained from empirical information; and, the highest variations were observed in the interception point. In this regard, the obtained slope in our work (−1.91) was very similar to those that were reported by García-Tejero et al. [31] (as it has been previously discussed), being the highest differences in the interception point. Similar findings were reported by Testi et al. [32] in pistachio trees, concluding that daily variations in net radiation resulted in parallel baselines, not being affected by the slopes.

Relating to the differences between T_TIC_ and T_SPH_, these would be related with three main questions: (i) the use of different thermal sensors, with different inherent characteristics; the monitoring area for a same tree is different (the angle of each sensor is different and for a same distance from the monitoring tree, the monitored area using the Flir One sensor is higher than for the case of Flir SC600; (ii) the operator to take the images was different; and, (iii) and the analysis software was not the same. These questions would explain the slight differences between the readings that were taken with both thermal cameras.

Once defined, the thermal indexes for the case of T_SPH_, high significant correlations were obtained as for T_SPH_, as for ∆T_SPH_ and CWSI. Even more, these relationships were especially representative for the case of T_SPH_ and CWSI vs. Ψ_leaf_ (0.81 and 0.73, respectively). 

For a proper management of deficit irrigation strategies, it is essential to identify the most appropriate and robust thermal index, as well as the best time of the day to perform the infrared thermal readings. In our case, thermal readings were taken at midday, as it was suggested by García-Tejero et al. [31] for this crop. The most significant differences in terms of T_SPH_, ∆T_SPH_, and CWSI, and the Ψ_leaf_ were detected, especially for the case of T_SPH_, and CWSI. If we consider the time-consuming aspect, then the absolute value of T_SPH_ would be the most recommendable because it is easy to calculate. The simplicity of this indicator could favor its usage as a preliminary indicator of stress. However, it can have major limitations for remote sensing of crop water status, whereas the CWSI would be more robust especially under more variable environmental conditions along the day.

## 5. Conclusions

Thermal imaging supposes a non-invasive technique in irrigated agriculture, easing the water resources management, irrigation scheduling, and the crop-water status monitoring. According to the obtained results of the present work, the Flir One sensor allows obtaining robust information, this being very similar to the obtained with other thermal sensors that were previously evaluated in several works. 

When considering the obtained relationships, CWSI would be the most appropriate thermal index to monitor the almond water status, although the absolute values of canopy temperature could offer previous information about crop-water status, if readings are taken under similar conditions.
